# Effects of strength training with elastic band programme on fitness components in young female handball players: a randomized controlled trial

**DOI:** 10.5114/biolsport.2022.106390

**Published:** 2021-07-15

**Authors:** Mehrez Hammami, Nawel Gaamouri, Herbert Wagner, Jeffrey C Pagaduan, Lee Hill, Pantelis T Nikolaidis, Beat Knechtle, Mohamed Souhaiel Chelly

**Affiliations:** 1 Research Unit (UR17JS01) « Sport Performance, Health & Society», Higher Institute of Sport and Physical Education of KsarSaîd, University of “La Manouba”, Tunis, Tunisia; 2 Higher Institute of Sport and Physical Education of Ksar Said, University of “La Manouba”, Tunis, Tunisia; 3 Department of Sport Science and Kinesiology, University of Salzburg, Salzburg, Austria; 4 School of Health Sciences, College of Health and Medicine, University of Tasmania, Newnham, Australia; 5 Division of Gastroenterology and Nutrition, Department of Pediatrics, McMaster University, Hamilton, Canada; 6 School of Health and Caring Sciences, University of West Attica, Athens, Greece; 7 Medbase St. Gallen Am Vadianplatz, St. Gallen, Switzerland; 8 Institute of Primary Care, University of Zurich, Zurich, Switzerland

**Keywords:** Repeated change of direction, Sprint, Jump, Skill, Team sports, Elastic band load capacity

## Abstract

This study examined the effect of a 10-week programme of strength training with elastic band (STEB) on fitness components in young female handball players. Twenty-six young female handball players (aged 15.8 ± 0.2 years) from the same club participated in this study. They were randomly assigned between experimental (EG; n = 13) and control (CG; n = 13) groups. The EG performed the STEB, replacing some handball-specific drills in the regular handball training. The CG followed the regular handball training (i.e., mainly technical-tactical drills, small sided and simulated games, and injury prevention drills). Two-way analyses of variance were used to assess: handgrip; back extensor strength; medicine ball throw; 30 m sprint times; Modified Illinois change-of-direction (Illinois-MT); four jump tests: squat jump (SJ), countermovement jump (CMJ), countermovement jump with arm swing (CMJA) and five-jump test (5JT); static (Stork test) and dynamic balance (Y Balance Test); and repeated sprint T-test (RSTT). Results revealed significant gains in handgrip - right (p < 0.001, d = 1.75: large), handgrip - left (p < 0.001, d = 2.52: large), back extensor (p < 0.001, d = 2.01: large), and medicine ball throw (p = 0.002, d = 0.95: large) with EG compared to the CG. The EG also demonstrated greater improvement in sprint performance over 20 m (Δ = 10.6%, p = 0.001, d = 1.07: large) and 30 m (Δ = 7.2%, p < 0.0001, d = 1.56: large) compared to the CG. The EG showed better Illinois-MT (Δ = 5.6%, p = 0.034, d = 0.62: medium) compared to the CG. Further, EG posted significant improvements in the SJ (Δ = 17.3%, p = 0.048, d = 0.58: medium), CMJ (Δ = 17.7%, p = 0.017 d = 0.71: medium), and CMJA (Δ = 16.3%, p = 0.019, d = 0.69: medium) compared to the CG. Similarly, the EG exhibited significant improvement in RSTT best time [p = 0.025, d = 0.66 (medium)], RSTT mean time [p = 0.019, d = 0.69 (medium)] and RSTT total time [p = 0.019, d = 0.69 (medium)] compared to the CG. In conclusion, the 10-week STEB improved the physical abilities in young female handball players.

## INTRODUCTION

Strength, sprint, change of direction and jump are critical factors to success in handball [1, 2]. In female handball, fast and dynamic movements consisting of accelerations, jumps, throws, change of directions, and hard body contacts are frequently interspersed with low intensity movements such as standing and walking [3, 4]. Therefore, efficient training approaches leading to the development of strength and power are essential to tolerate physical demands and prevent injuries in female handball [4, 5].

Studies support the utility of strength training schemes in performance enhancement of female handball athletes. For example, Hammami et al. [6] revealed that strength training combined with plyometric training facilitated improvement in physical abilities of U14 female handball players. Another investigation found amelioration in sprint, change of direction, vertical and horizontal jump, strength, and repeated change of direction performances after 8-week complex training in U17 female handball players [6]. Moreover, Chaabene et al. [7] revealed that 8-week eccentric hamstring training enhanced linear sprint time, change of direction, jump, and repeated sprint performance in young female handball players. In addition, Ignjatovic et al. [8] suggested that 12-week medicine ball training, when incorporated into a regular training session, demonstrated sport-specific training improvement in the upper body for young female handball players.

Recently, strength training using an elastic band (STEB) has been used as an alternative strength training scheme to improve physical performance in handball [9–11]. STEB is affordable, easy to use, portable, and provides a safe and effective progressive overload technique, applicable not only to athletes, but also to injured patients and sedentary people. In addition, STEB is a time-saving method for improving muscle strength and power of athletes during physical preparation [9]. In contrast, quantification of training load STEB is difficult to distinguish. A few studies have explored STEB in handball [9, 10, 12]. For example, Anderson et al. [10] demonstrated that STEB, incorporated into the regular handball training sessions, improved explosive lower-limb performance in young female handball players compared to handball training alone. Similarly, Mascarin et al. [13] observed enhancement in athletic performance, external rotator muscle strength, and balance after 6 weeks of STEB in young female handball players.

Given the potential of STEB in development of physical capabilities in handball, there seems to be a paucity in the literature investigating STEB in youth female handball athletes. Such undertaking can provide useful information in the application of STEB in the female youth handball setting. Thus, this study aimed to examine the effects of ten-week STEB on upper limb strength performances, sprint, change of direction, repeated change of direction, balance and jump performances in young female handball players. It is hypothesized that STEB improves upper limb power performances, sprint, change of direction, repeated change of direction and jump performances in young female handball players.

## MATERIALS AND METHODS

### Ethical approval

All procedures were approved by the local ethical committee for the use of human participants of the Higher Institute of Sports and Physical Education of Ksar Saïd, Tunisia. The study was conducted in accordance with the latest version of the Declaration of Helsinki. Written informed parental consent (for those < 18 years) and participants’ assent were obtained prior to the start of the study. All participants and their parents/legal representatives were fully informed about the experimental protocol and its potential risks and benefits.

### Participants

Twenty-six young female handball players from the same club participated in this study. They were randomly assigned between an elastic band training group (experimental group) (EG; n = 13; age = 15.7 ± 0.2 years; body mass = 64 ± 3 kg; height = 1.70 ± 0.04 m; % body fat = 25.3 ± 1.7; and maturity-offset = 3.3 ± 0.4 years) and a control group of players who maintained their standard in-season regimen (CG; n = 13; age = 15.8 ± 0.2 years; body mass = 64 ± 4 kg; height = 1.67 ± 0.04 m; % body fat = 26.6 ± 3.4 ; and maturity-offset = 3 ± 0.3 years). All participants were involved in five to six training sessions per week (90–120 min each session). The EG performed the elastic band training programme in replacement of some handball-specific drills so that the overall training volume was similar between groups.

The study was conducted to examine the effect of a 10-week STEB programme on fitness components in young female handball players. The training intervention was conducted during the in-season period in the year 2018–2019. In the week before the intervention, two, 90-min sessions were administered to allow player familiarization with the test procedures. Measurements were made in a fixed order over four days, immediately before and four days after the last strength training session. Subjects did not participate in any exhausting exercise 24 hours before testing, and no food or caffeine-containing drinks were taken for two hours before testing. A standardized warm-up (10–20 min of low- to moderate-intensity aerobic exercise and dynamic stretching) preceded all the tests. On the first test day, sprinting and change of direction abilities were measured. The second day was devoted to jumping and handgrip strength assessments. On the third day, anthropometric measurements were administered. After that, back extensor strength and medicine ball throw tests were conducted. On the fourth day, the athletes completed the balance and repeated sprint tests.

### Procedures and evaluation

#### Day one

##### 30 m sprint performance

Players started from a split stance standing position, with the front foot 0.2 m from the first photocell beam and sprinted for 30 m on command. Split times for 5, 10, 20 and 30 m distances were recorded for analysis [6].

##### Modified Illinois change-of-direction test (Illinois-MT)

Four cones formed the change-of-direction area for the modified Illinois test [14]. On command, players sprinted 5 m, turned and ran back to the starting line, then, swerving in and out of the 4 markers, completed two, 5 m sprints sprints. No advice was given as to the most effective technique, but players were instructed to complete the test as quickly as possible without cutting over markers. A trial is repeated if an athlete ‘cuts’ a marker while completing the task. Three trials were allowed for the 30 m sprint performance and Illinois-MT (separated by 6–8 min of recovery) and the best time performances were noted using paired photocells (Microgate, Bolzano, Italy).

#### Day two

##### Vertical jump

Jump height was assessed using an infrared photocell mat connected to a digital computer (Optojump System; Microgate SARL) that measured contact and flight times and the height of jump with a precision of 1/1000 seconds [15]. Participants began the squat jump (SJ) at a knee angle of ~90°, avoiding any downward movement, and pushed upward, keeping their legs straight throughout. The countermovement jump (CMJ) began from an upright position; a rapid downward movement to a knee angle of ~90° (again self-controlled, using a mirror) accompanied the beginning of the push-off. During the countermovement jump with arm swing (CMJA), with hands used freely while jumping. Three trials were executed for each jump test, with one minute rest in between trials, and the highest jump from each test utilized in subsequent analyses.

##### Five-jump test (5JT)

The test was performed as previously described [6]. From an upright standing position with both feet flat on the ground, participants tried to cover as much distance as possible with 5 forward jumps, alternating left- and right-leg ground contacts. Participants were allowed 3 maximal trials, with 3 minutes of rest between efforts, and the best performance was used for analyses [6].

##### Handgrip strength test

The hand dynamometer (Takei, Tokyo, Japan) was held with the arm at a right angle and the elbows at the side of the body [16]. The instrument was adjusted so that its base rested on the first metacarpal and the handle rested on the middle of the 4 fingers. A maximal isometric effort was maintained for 5 seconds, without ancillary body movements. Two trials were administered for each hand, with 1 minute of rest between trials, and the highest readings were used in subsequent analyses.

#### Day three

##### Anthropometry

Anthropometric measurements included height and sitting height (accuracy of 0.1 cm; Holtain Q 3, United Kingdom) and body mass (0.1 kg; Tanita BF683W scales, Munich, Germany). The overall percentage of body fat was estimated from biceps, triceps, subscapular, and suprailiac skinfolds, using the equations of Durnin and Womersly [17] for children and youth females:

% Body fat = (495/ D) - 450

where D = 1.1369–0.0598 (log sum of 4 skinfolds)

Maturity status was calculated using the equation of Mirwald et al. [18], an approved non-invasive method to predict years from peak height velocity:

Maturity offset = −9.38 + (0.000188 × leg length × sitting height) + (0.0022 × age × leg length) + (0.00584 × age × sitting height) + (0.0769 × weight/height ratio)

##### Back extensor strength

Maximal isometric back extensor strength was measured using a back extensor dynamometer (Takei) [19]. Participants stood on the dynamometer, with their feet shoulder width apart and gripped the handle bar positioned across the patellae. The chain length was adjusted so that initially the legs were held straight and the back was flexed to 30°, as guided by wall markings. Participants then stood upright without bending their knees, pulling upward as strongly as possible.

##### Medicine ball throw

The test was performed using 21.5-cm diameter, 3-kg rubber medicine balls (Tigar, Pirot, Serbia) powdered with magnesium carbonate. A familiarization session included a brief description of the optimal technique [20]. The seated player grasped the medicine ball with both hands, and on a signal forcefully pushed the ball from the chest. The score was measured from the front of the sitting line to the powder-marked spot where the ball landed.

#### Day four

##### Stork balance test

To perform the Stork balance test, participants stood with their opposite foot against the inside of the supporting knee and both hands on the hips. On the command, participants raised the heel of their foot from the floor and attempted to maintain their balance as long as possible. The trial ended if the participant either moved her hands from her hips, the ball of the dominant foot moved from its original position, or if the heel touched the floor. This test was carried out on both legs. The test was timed (s) using a stopwatch. The recorded score (duration in seconds) was the best of three attempts [21].

##### Dynamic balance test

Dynamic balance was assessed on the dominant leg, using the Y-balance test [21]. Supine leg lengths were first determined from the anterior superior iliac spine to the most distal aspect of the medial malleolus. Subjects then stood barefoot and single-legged, with the tip of their great toe at the centre of the grid, and reached in anterior, postero-medial and postero-lateral directions, marked on the floor by tape. The posterior lines extended at an angle of 135° from the anterior line. Trials were repeated if the participant (1) did not touch the required line with the reaching foot while maintaining weight bearing on the stance leg, (2) lifted the stance foot from the centre of the grid, (3) lost balance, (4) did not maintain start and return positions for one full second, or (5) touched the reaching foot to gain support. The maximal reach was measured in each direction, and a composite score calculated as [maximum anterior + maximum postero-medial + maximum postero-lateral reach distance]/[leg length × 3] × 100). [21] Three trials were conducted in each direction, with two-minute rest intervals.

##### Repeated sprint T-test (RSTT)

This test offers a reliable and valid measurement [22] of the ability to change directions rapidly, simulating a game with short, intense efforts, recovery periods and multi-directional displacements. Seven executions of the agility T-test were made, with subjects walking back slowly to the next start point during 25 s recovery intervals. Measures included best time (BT), mean time (MT), total time (TT) and a fatigue index calculated as [23]:

FI = ((total time / (best time × 7)) × 100) – 100

##### Strength training programme

The training intervention consisted of a progressive 10-week upper and lower STEB programme. STEB was completed during the mid-portion of the competitive season 2018/2019 (from January to March). The design of the STEB intervention was based on the players’ previous training records and research results [9, 10, 12, 13] (Table 1). Bi-weekly STEB sessions (Tuesdays and Thursdays) included four exercises for the upper limb and four exercises for the lower limb. The elastic band (Thera-Bands; Hygenic Corporation, Akron, OH, USA) system includes 4 latex bands of differing elasticity: red (250% elongation (3.2 kg)), green (250% elongation (4.4 kg)), blue (250% elongation (6 kg)) and black (250% elongation (8 kg)). Upper limb exercises included flies, row with high elbows, trunk rotation, and standing press. Lower limb exercises involved knee extension, knee flexion, half squat, and hip adduction. The order of exercises was alternated (upper limb exercise then lower limb exercise). The elastic band was folded to double its resistance to extension in the lower limb exercise, but not double for the upper limb exercise. The necessary amplitudes of movement during each exercise were calculated individually, thus determining appropriate attachments of the bands to the wall and the player’s body. Recovery between sets was 30 seconds. All exercises were performed with the maximal effort level. The initial length of the elastic band was 120 cm for all exercises. The STEB was not added to the regular handball training but was immediately performed after the warm-up programme [10] in replacement of some low-intensity technical-tactical handball drills. The STEB replacement activity accounted for < 10% of the total handball-training load (competitive and friendly matches not accounted for). The CG subjects followed their regular handball training (i.e., mainly technical-tactical drills, small sided and simulated games, and injury prevention drills). The overall handball training load was comparable between groups (using the Borg Rating of Perceived Exertion (RPE)). This is because they were following similar handball training routines consisting of 6 sessions per week with 90 to 120 min each.

**TABLE 1:** Strength training programme.

Exercises	Week 1	Week 2	Week 3	Week 4	Week 5	Week 6	Week 7	Week 8	Week 9	Week 10
**Upper limb**	Red elastic band at 250% elongation (3.2 kg)	Green elastic band at 250% elongation (4.4 kg)			Blue elastic band at 250% elongation (6 kg)			Black elastic band at 250% elongation (8 kg)		

	Sets × RepsSets	× RepsSets	× RepsSets	× RepsSets	× RepsSets	× RepsSets	× RepsSets	× RepsSets	× RepsSets	× Reps
Flies	3 × 10	3 × 10	4 × 10	5 × 10	3 × 10	4 × 10	5 × 10	3 × 10	4 × 10	5 × 10
Row with high elbows	3 × 10	3 × 10	4 × 10	5 × 10	3 × 10	4 × 10	5 × 10	3 × 10	4 × 10	5 × 10
Trunk rotation	3 × 10	3 × 10	4 × 10	5 × 10	3 × 10	4 × 10	5 × 10	3 × 10	4 × 10	5 × 10
Standing press	3 × 10	3 × 10	4 × 10	5 × 10	3 × 10	4 × 10	5 × 10	3 × 10	4 × 10	5 × 10

**Lower limb**	Red elastic band «Folding» at 250% elongation (6.4 kg)	Green elastic band «Folding» at 250% elongation (8.8 kg)			Blue elastic band «Folding» at 250% elongation (12 kg)			Black elastic band «Folding» at 250% elongation (16 kg)		

	Sets × RepsSets	× RepsSets	× RepsSets	× RepsSets	× RepsSets	× RepsSets	× RepsSets	× RepsSets	× RepsSets	× Reps
Knee extension	3 × 10	3 × 10	4 × 10	5 × 10	3 × 10	4 × 10	5 × 10	3 × 10	4 × 10	5 × 10
Knee flexion	3 × 10	3 × 10	4 × 10	5 × 10	3 × 10	4 × 10	5 × 10	3 × 10	4 × 10	5 × 10
Half squat	3 × 10	3 × 10	4 × 10	5 × 10	3 × 10	4 × 10	5 × 10	3 × 10	4 × 10	5 × 10
Hip adduction	3 × 10	3 × 10	4 × 10	5 × 10	3 × 10	4 × 10	5 × 10	3 × 10	4 × 10	5 × 10

N.B.: the overall handball training load was comparable between the groups (using the Borg Rating of Perceived Exertion (RPE)).

### Statistical analyses

Statistical analyses were carried out using the SPSS 20 program for Windows (United States, Armonk, NY: IBM Corp). Normality of all variables was tested using the Kolmogorov–Smirnov test procedure. Data are presented as mean (SD), and as median values for skewed variables. Independent sample t tests were performed separately, to determine pre-intervention and post-intervention changes for the experimental and control groups, with the magnitude of the changes determined via Cohen d effect sizes [24]. Training-related effects were assessed by 2-way analyses of variance (group × time). The criterion for statistical significance was set at p < 0.05, whether a positive or a negative difference was seen (i.e., a 2-tailed test was adopted). The reliabilities of all dependent variables were assessed by calculating intraclass correlation coefficients (2-way mixed) [25]. Effect sizes were determined by converting partial eta-squared to Cohen d [24]; values were classified as small (0.00 ≤ d ≤ 0.49), medium (0.50 ≤ d ≤ 0.79), and large (d ≥ 0.80).

## RESULTS

Test-retest reliability was above the established threshold and ranged from 0.742 to 0.992 according to the intra-class correlation coefficient and ranged from 2.0 to 54.1 according to the coefficient of variation (Table 2). Initial values showed no significant intergroup differences for any of the dependent variables. All data for both groups were significantly increased after the 10-week (Table 3 and Table 4) intervention with the exception of the Stork balance test (left leg), which remained unchanged for the control group (Table 4). With a significant group × time interaction, the EG showed enhancement of handgrip right [p < 0.001, d = 1.75 (large)], handgrip left [p < 0.001, d = 2.52 (large)], back extensor strength [p < 0.001, d = 2.01 (large)] and medicine ball throw [p = 0.002, d = 0.95 (large)] compared to the CG (Table 4). The EG significantly improved sprint performance at 20 m [Δ = 10.6%, p = 0.001, d = 1.07 (large)] and 30 m [Δ = 7.2%, p < 0.0001, d = 1.56 (large)] distances compared to the CG (Table 5). No significant differences in 5 m and 10 m sprint ability were found in the EG and CG. Enhancement in the Illinois-MT was also demonstrated by the EG [Δ = 5.6%, p = 0.034, d = 0.62 (medium)] in comparison with the CG (Table 5). Moreover, the EG showed significantly increased SJ [Δ = 17.3%, p = 0.048, d = 0.58 (medium)], CMJ [Δ = 17.7%, p = 0.017 d = 0.71 (medium)], and CMJA [Δ = 16.3%, p = 0.019, d = 0.69 (medium)] compared to the CG. No significant difference was found in 5JT between the EG and CG. Similarly, significant group × time interactions favouring EG than CG existed at RSTT-BT [p = 0.025, d = 0.66 (medium)], RSTT-MT [p = 0.019, d = 0.69 (medium)], and RSTT-TT [p = 0.019, d = 0.69 (medium)] (Table 4). There were no significant differences in group × time interaction during static and dynamic balance performance between the EG and CG (Table 4).

**TABLE 2:** Reliability and coefficient of variation of performance tests.

	ICC	95%CI	CV
5m	0.985	0.966–0.993	4.1
10m	0.970	0.934–0.987	2.4
20m	0.883	0.740–0.948	2
30m	0.913	0.805–0.961	2
Illinois-MT	0.887	0.748–0.949	2.3
SJ	0.954	0.897–0.979	8.8
CMJ	0.947	0.881–0.976	8.2
CMJA	0.943	0.873–0.975	8
5JT	0.992	0.981–0.996	10.5
Handgrip - right	0.896	0.769–954	7.2
Handgrip - left	0.924	0.830–0.966	7.9
Back extensor	0.939	0.864–0.973	7.9
Medicine ball throw	0.986	0.970–0.994	16.8
Stork right	0.763	0.471–0.894	45.5
Stork left	0.742	0.424–0.884	54.1
RL/L	0.954	0.897–0.979	10
RL/B	0.951	0.891–0.978	9.2
RL/R	0.954	0.898–0.980	19.1
LL/R	0.961	0.913–0.982	11.4
LL/B	0.913	0.806–0.961	8.6
LL/L	0.828	0.616–0.923	17.7

Values are mean ± SD. Abbreviations: CI = confidence intervals; CV = coefficient of variation; CMJ = counter-movement jump; CMJA = counter-movement jump; ICC = intraclass correlation coefficient; Illinois-MT = Illinois modified test; SJ = squat jump; B = background; L = left; R = right; LL = left leg; RL = right leg

**TABLE 3:** Upper-limb performance in experimental and control groups before and after the 10-week intervention.

	Experimental group (n = 17)					Control group (n = 17)					ANOVA group x time interaction	

	Pre	Post	%Δ change	Paired t test		Pre	Post	%Δ change	Paired t test		p	Cohen d
				p	d (Cohen)				p	d (Cohen)		
Handgrip - right (N)	227 ± 9	297 ± 18	31.0 ± 6.8	p< 0.001	-5.12	229 ± 22	238 ± 21	3.8 ± 1.7	p< 0.001	-0.44	p< 0.001	1.75

Handgrip - left (N)	207 ± 17	292 ± 16	41.2 ± 5.1	p< 0.001	-5.36	222 ± 14	235 ± 12	5.9 ± 2.9	p< 0.001	-1.04	p< 0.001	2.52

Back extensor (N)	753 ± 55	1175 ± 139	56.1 ± 14.2	p< 0.001	-4.16	793 ± 62	866 ± 80	9.3 ± 6.6	p< 0.001	-1.06	p< 0.001	2.01

Medicine ball throw (m)	3.0 ± 0.4	4.1 ± 0.4	40.1 ± 10.3	p< 0.001	-2.86	3.1 ± 0.6	3.3 ± 0.6	6.7 ± 7.8	0.007	-0.35	0.002	0.95

Values are mean ± SD.

**TABLE 4:** Lower-limb performance in experimental and control groups before and after the 10-week intervention.

	Experimental group (n = 17)					Control group (n = 17)					ANOVA group x time interaction	

	Pre	Post	%Δ change	Paired t test		Pre	Post	%Δ change	Paired t test		p	Cohen d
				p	d (Cohen)				p	d (Cohen)		
Sprint												
5m (s)	1.28 ± 0.05	1.16 ± 0.05	9.8 ± 1.9	p< 0.001	2.50	1.28 ± 0.06	1.19 ± 0.03	6.8 ± 4.5	p< 0.001	1.97	0.174	0.39
10m (s)	2.24 ± 0.05	2.09 ± 0.05	6.9 ± 1	p< 0.001	3.12	2.24 ± 0.06	2.12 ± 0.04	5.2 ± 2.6	p< 0.001	2.45	0.200	0.37
20m (s)	3.76 ± 0.10	3.36 ± 0.09	10.6 ± 1.1	p< 0.001	4.38	3.75 ± 0.05	3.53 ± 0.07	5.8 ± 2.5	p< 0.001	3.76	0.001	1.07
30m (s)	5.38 ± 0.10	4.99 ± 0.08	7.2 ± 1.2	p< 0.001	4.48	5.54 ± 0.05	5.40 ± 0.09	2.5 ± 1.6	p< 0.001	2.00	p< 0.001	1.56

Change of direction												
Illinois-MT	13.14 ± 0.22	12.41 ± 0.20	5.6 ± 0.3	p< 0.001	3.61	13.17 ± 0.37	12.73 ± 0.11	3.3 ± 2.3	p< 0.001	1.68	0.034	0.62
Jump												
SJ (cm)	22.4 ± 1.6	26.2 ± 1.3	17.3 ± 6.1	p< 0.001	-2.71	22.7 ± 2.4	24.4 ± 1.9	8 ± 5.8	p< 0.001	-0.82	0.048	0.58
CMJ (cm)	23.4 ± 1.6	27.5 ± 2	17.7 ± 2.7	p< 0.001	-2.36	23.9 ± 2.2	25.3 ± 2	6.1 ± 6.2	0.002	-0.69	0.017	0.71
CMJA (cm)	24.7 ± 1.6	28.7 ± 1.8	16.3 ± 2.7	p< 0.001	-2.44	25.2 ± 2.3	26.6 ± 1.8	6.2 ± 8.1	0.011	-0.71	0.019	0.69
5JT (m)	7.9 ± 0.9	8.4 ± 0.9 (8.30) ^a^	7.5 ± 4.6	p< 0.001	-0.58	8.1 ± 1.6	8.4 ± 1.5 (8.50) ^a^	4.5 ± 3.2	p< 0.001	-0.20	0.300	0.30

RSTT												
RSTT-BT (s)	12.60 ± 0.19	12.10 ± 0.22	4 ± 0.8	p< 0.001	2.53	12.63 ± 0.18	12.37 ± 0.13	2.1 ± 0.6	p< 0.001	1.72	0.025	0.66
RSTT-MT (s)	12.92 ± 0.18	12.36 ± 0.24	4.3 ± 0.8	p< 0.001	2.75	12.92 ± 0.18	12.66 ± 0.13	2.1 ± 0.6	p< 0.001	1.72	0.019	0.69
RSTT-TT (s)	90.42 ± 1.27	86.51 ± 1.65	4.3 ± 0.8	p< 0.001	2.76	90.5 ± 1.25	88.6 ± 0.88	2.1 ± 0.6	p< 0.001	1.83	0.019	0.69
RSTT-FI (%)	2.49 ± 0.42 (2.46) ^a^	2.13 ± 0.81	14.3 ± 29.9	0.107	0.58	2.30 ± 0.03	2.35 ± 0.02	2.2 ± 0.7	p< 0.001	-2.04	0.107	0.47

Y Balance test Right support leg												
RL/L (cm)	74 ± 7	80 ± 5	8 ± 7.7	0.001	-1.03	75 ± 8	82 ± 8	9.1 ± 7.6	p< 0.001	-0.91	0.794	0.06
RL/B (cm)	85 ± 8	94 ± 8	10.7 ± 4.8	p< 0.001	-1.17	89 ± 8	94 ± 8	5 ± 2.5	p< 0.001	-0.65	0.314	0.29
RL/R (cm)	46 ± 10	51 ± 9	13.2 ± 10.8	p< 0.001	-0.55	50 ± 8	56 ± 6	14.3 ± 20.9	0.007	-0.88	0.895	0.06

Left support leg												
LL/R (cm)	78 ± 10	84 ± 9	8.3 ± 4.8	p< 0.001	-0.66	80 ± 8	86 ± 6	8.2 ± 10.8	0.007	-0.88	0.961	0.06
LL/B (cm)	96 ± 9	101 ± 9	5.3 ± 4	p< 0.001	-0.58	95 ± 8	99 ± 7	3.9 ± 2.9	p< 0.001	-0.55	0.761	0.08
LL/L (cm)	46 ± 9	52 ± 8	15.7 ± 13.3	p< 0.001	-0.73	45 ± 8	49 ± 8	10.1 ± 8	p< 0.001	-0.52	0.612	0.14

Stork balance test												
RL (s)	2.67 ± 1.20 (2.48) ^a^	3.92 ± 1.40	56.6 ± 40.1	p< 0.001	-1.00	2.72 ± 1.30 (1.98) ^a^	3.95 ± 2.04	53 ± 51.4	0.015	-0.75	0.987	0.000
LL (s)	2.71 ± 1.47	3.36 ± 1.71	42.7 ± 66.2	0.111	-0.42	2.46 ± 1.37	3.29 ± 1.48	48.2 ± 59.3	0.090	-0.61	0.823	0.001

Values are mean ± SD. Abbreviations: ^a^ Median reported for rightward skewing data; Illinois-MT = Illinois modified test; SJ = squat jump; CMJ = countermovement jump; CMJA = countermovement jump with arms; 5JT = 5 jump test; RSTT = repeated sprint T-test; BT = best time; MT = mean time; TT = total time; FI = fatigue index; RL = right leg; L = left; R = right; B = background; LL = left leg.

## DISCUSSION

This study examined the effects of a 10-week in-season STEB programme on components of physical performance in junior female handball players. Novel findings in this study include various adaptations in sprint performance (only 20 m and 30 m distances) and non-significant changes in balance tasks with STEB. Other findings demonstrated equivocal results in sprint, change of direction, and jump performances after STEB. STEB also demonstrated improvement in upper limb performance indices.

Novel findings on the effects of STEB on sprint performance were identified in this study. First, the present study showed improvement in 20 m (p = 0.001; d = 1.07: large) and 30 m (p < 0.001; d = 1.56: large) sprint performances after STEB. The results agreed with previous studies [9, 26]. High levels of linear speed over short and medium (< 30-m) distances are important physical fitness attributes in handball [1] Enhancement in sprint performance after STEB can be attributed to transference capability of STEB to maximal running from increased knee extensor and flexor power production [26–28]. In addition, the amelioration in maximal speed from STEB may be influenced by neural adaptations because less hypertrophy occurs after training with elastic bands [29]. Secondly, sprint ability in 5 m and 10 m were not affected by STEB. Initial acceleration (over 5 and 10 m) has proven more difficult to enhance than maximal velocity, probably due to the smaller margin for improvement and various mechanical forces involved [6]. The lack of improvement in the acceleration phase after STEB in this study contrasts with the study by Aloui et al. [9]. The discrepancy in results could be explained by differences in the intensity of the training programme and also by gender training adaptation. More studies are needed to elucidate physiological mechanisms responsible for sprint performance outcomes with STEB.

This is the first study to investigate the effects of STEB on the balance performance in female handball players. Although improvement in static and dynamic balance was detected with STEB, this change was not significantly different compared to the control group. The results partially contradicted the findings by Kordi et al. [30], which exhibited increased static balance performance after a 12-week STEB in children with developmental coordination disorder. It may be possible that non-transference of neuromuscular adaptation achieved from STEB is present in performance of balance tests. In addition, inclusion of a 5-minute preventive exercises during warm-up for both groups may have influenced the lack of a significant difference between groups.

The 10-week STEB also demonstrated enhancement in agility performance. Rapid change-of-direction tasks are critical in handball [31]. The results of Wagner et al. 2019 [32] indicated the importance of specific agility both in offense and defence, in throwing velocity in the jump shot as well as in aerobic performance, to become a world-class adult female team handball player. Our results showed improved agility performance after STEB (p = 0.034; d = 0.62: medium). This was in accordance with the findings of Aloui et al. [9] after 8-week STEB. Conversely, the findings of this study contradicted the results reported by Anderson et al. [10], who found no significant change in agility after 6-week STEB in young female handball plyers. The discrepancy in results with the previous study could be explained by the variability in test procedures and interventions (frequency, duration, and progression of training relative to the playing season). The STEB in this study probably affected the velocity factor of the power output more than the force factor for the lower limbs. This is supported by an increased between-period difference velocity in the change of direction performance. Greater force is generated during each repetition during the last half of the concentric action and the first half of the eccentric action with STEB exercises, thereby developing concentric to eccentric contraction transition properties [26]. In this study, STEB exhibited various characteristics in change of direction, pertaining to the repeated agility task. Repeated high-intensity agility is dependent on neuromuscular (e.g., neural drive and motor-unit activation) and metabolic factors (e.g., oxidative capacity, creatine phosphate recovery and H+ buffering) [33]. The current results showed increased RSTT-BT (p = 0.025; d = 0.66: medium), RSTT-MT (p = 0.019; d = 0.69: medium), and RSTT-TT (p = 0.019; d = 0.69: medium) after STEB. These results are in line with the findings of Aloui et al. [9]. Improvement in repeated agility may be related to neural adaptations. No significant change in RSTT-FI was identified after STEB. One possible explanation for this result is the poor reproducibility of this particular measure [34].

In this study, the STEB demonstrated contrasting results in jump performances. Firstly, it exhibited increased SJ (p = 0.048; d = 0.58: medium), CMJ (p = 0.017; d = 0.71: medium) and CMJA (p = 0.019; d = 0.69: medium). The STEB may have affected the velocity factor of the power output more than the force factor for the lower limbs. This is supported by the velocity data, which showed an increased between-period difference in velocity in the vertical jump performance [9]. The mechanism responsible for this effect has been attributed mainly to neural adaptations such as an increased nerve conduction velocity, maximization of the electromyogram, improved intermuscular coordination, an enhanced motor unit recruitment strategy, and increased excitability of the Hoffman reflex (H-reflex), as well as changes in muscle size and architecture, in the mechanical characteristics of the muscle-tendon complex, and changes in single-fibre mechanics [29]. The SJ, CMJ, and CMJA findings agree with the results reported by Anderson et al. [10]. However, the SJ and CMJ results in this study contrasted with the findings of Aloui et al. [9], which demonstrated no significant changes in SJ and CMJ indices after 8-week STEB in elite junior male handball players. Discordant outcomes from the previous study may be related to methodological differences. Another finding in this study was the non-significant difference in 5JT. The STEB may not be sufficient in delivering strength, flexibility, and coordination of the upper and lower limbs specific to the physiological demands of 5JT. Further studies are needed to elucidate factors differentiating jump performance capacities with STEB.

The STEB increased the upper limb strength and power indices in female handball athletes. A high level of upper limb power (for passing and throwing the ball) is an important factor in handball performance. [2] The results of the current study indicated increased handgrip-right (p < 0.001; d = 1.75; large), handgrip-left (p < 0.001; d = 2.52; large), back extensor (p < 0.001; d = 2.01; large), and medicine ball throw (p < 0.001; d = 0.95; large) after STEB. In a similar study, Anderson et al. [10] demonstrated improvement in ball throwing velocity and bench press performance after 6-week STEB. Mascarin et al. [12] recorded enhancement in average power value of shoulder internal, ball throwing speed from standing and jumping, after 6-week STEB in young female handball players. In another study by Mascarin et al. [13], STEB developed muscular strength of external rotator muscles and muscular balance in female youth handball players. The enhancement of upper limb performance parameters in this study can be linked to an increased rate of force development from greater motor recruitment, [35, 36] tendon stiffness, [37] or fascicle length [38, 39].

This study has some limitations that need to be acknowledged. First, no direct physiological (e.g., electromyography; isokinetic strength test) or biomechanical (e.g., vertical ground reaction force) measures were conducted. The aforementioned measures have to be considered in future research. Second, the Stork balance test result should be interpreted with caution as the results presented low reliability for our study population (ICC = 0.763 and ICC = 0.742 for the right and the left leg respectively). Lastly, addition of a strength training group for comparison with STEB would provide more useful information in the utilization of STEB.

## CONCLUSIONS

Physical conditioning is an essential intervention for improving strength and power, related to physical abilities crucial for youth handball performance. In this study, the STEB used as a physical conditioning scheme among young female handball players increased strength and power, accompanied by improvement in sprint, jump and change of direction. Thus, administration of a twice-weekly, 35-minute STEB can be utilized as an alternative to improve the physical qualities in young female handball athletes.
